# Supplementary material for: Palliative Quad Shot Radiation Therapy with or without Concurrent Immune Checkpoint Inhibition for Head and Neck Cancer
Source: Cancers (Basel). 2024 Mar 5;16(5):1049. doi: 10.3390/cancers16051049 (PMC10931206; doi:10.3390/cancers16051049)
Supplement: Supplementary file 1 [file cancers-16-01049-s001.zip › cancers-2904186-supplementary.pdf]

Supplementary Table S1. Univariate and multivariable analyses of factors affecting distant control.

	Univariate analysis		Multivariable analysis	
	HR (95% CI)	p-value	HR (95% CI)	p-value
Age	0.972 (0.944-1.002)	.068	0.983 (0.951-1.017)	.333
Race	0.895 (0.364-2.198)	.809	Not included	
Sex	0.764 (0.289-2.019)	.587	Not included	
Primary site	1.015 (0.905-1.138)	.802	Not included	
T-stage	0.988 (0.606-1.610)	.962	Not included	
N-stage	1.416 (0.930-2.155)	.105	1.421 (0.905-2.231)	.127
M-stage	2.032 (0.941-4.388)	.071	1.645 (0.708-3.820)	.247
Smoking	1.563 (0.862-2.835)	.142	1.552 (0.808-2.979)	.187
ECOG PS ≥2	0.923 (0.401-2.123)	.850	Not included	
Surgery	1.355 (0.617-2.975)	.449	Not included	
Prior ST	1.549 (0.676-3.546)	.301	Not included	
Conc QS+ICI	1.208 (0.560-2.608)	.630	Not included	
Prior RT	1.030 (0.484-2.192)	.939	Not included	
Adjuvant ICI	1.509 (0.660-3.448)	.329	Not included	
No. of QS cycles	0.523 (0.206-1.329)	.173	0.671 (0.257-1.755)	.416

Abbreviations: HR = hazard ratio; CI = confidence intervals; ECOG PS = Eastern Cooperative Oncology Group performance status; ST = systemic therapy; conc = concurrent; QS = ‘Quad Shot’; ICI = immune checkpoint inhibitor; RT = radiation therapy.

Supplementary Table S2. Univariate and multivariable analyses of factors affecting overall survival.

	Univariate analysis		Multivariable analysis	
	HR (95% CI)	p-value	HR (95% CI)	p-value
Age	0.990 (0.970-1.011)	.359	Not included	
Race	1.152 (0.619-2.144)	.655	Not included	
Sex	0.950 (0.489-1.847)	.880	Not included	
Primary site	0.903 (0.829-0.984)	.020*	0.925 (0.843-1.014)	.097
T-stage	0.919 (0.672-1.258)	.599	Not included	
N-stage	1.014 (0.773-1.331)	.920	Not included	
M-stage	0.563 (0.283-1.121)	.102	0.833 (0.398-1.746)	.629
Smoking	1.495 (1.004-2.226)	.048*	1.342 (0.882-2.040)	.170
ECOG PS ≥2	2.198 (1.252-3.856)	.006*	1.844 (1.020-3.334)	.043*
Surgery	1.417 (0.815-2.462)	.217	Not included	
Prior ST	0.980 (0.569-1.686)	.941	Not included	
Conc QS+ICI	1.053 (0.615-1.805)	.850	Not included	
Prior RT	0.892 (0.526-1.515)	.673	Not included	
Adjuvant ICI	0.865 (0.499-1.501)	.607	Not included	
No. of QS cycles	0.937 (0.548-1.603)	.813	Not included	

Abbreviations: HR = hazard ratio; CI = confidence intervals; ECOG PS = Eastern Cooperative Oncology Group performance status; ST = systemic therapy; conc = concurrent; QS = ‘Quad Shot’; ICI = immune checkpoint inhibitor; RT = radiation therapy.

* Considered statistically significant based on *P*-value <.050.
